# Age-standardized incidence, mortality rate, and trend changes of thyroid cancer in the Balearic Islands during the 2000–2020 period: a population-based study

**DOI:** 10.1530/ETJ-22-0183

**Published:** 2023-04-21

**Authors:** Santiago Tofé, Iñaki Argüelles, Ana Forteza, Cristina Álvarez, Alessandra Repetto, Luis Masmiquel, Irene Rodríguez, Eladio Losada, Nuria Sukunza, María Cabrer, Mildred Sifontes, María del Mar del Barrio, Antonia Barceló, Álvaro Tofé, Vicente Pereg

**Affiliations:** 1 Department of Endocrinology, University Hospital Son Espases, Palma de Mallorca, Spain; 2 Department of Pathology, University Hospital Son Espases, Palma de Mallorca, Spain; 3 Department of Surgery, Section of Endocrine Surgery, University Hospital Son Espases, Palma de Mallorca, Spain; 4 Department of Nuclear Medicine, University Hospital Son Espases, Palma de Mallorca, Spain; 5 Department of Endocrinology, University Hospital Son Llatzer, Palma de Mallorca, Spain; 6 Department of Endocrinology, Hospital Can Misses, Ibiza, Spain; 7 Department of Endocrinology, Hospital de Manacor, Manacor, Spain; 8 Department of Endocrinology, Hospital Comarcal de Inca, Inca, Spain; 9 Department of Pathology, Hospital Mateu Orfila, Menorca, Spain; 10 Department of Laboratory, University Hospital Son Espases, Palma de Mallorca, Spain; 11 Department of Maxillofacial Surgery, Hospital Puerta del Mar, Cádiz, Spain

**Keywords:** age-standardized incidence, thyroid cancer, mortality, overdiagnosis

## Abstract

**Objective:**

Global thyroid cancer (TC) incidence is growing worldwide, but great heterogenicity exists among published studies, and thus, population-specific epidemiological studies are needed to adequate health resources and evaluate the impact of overdiagnosis.

**Methods:**

We conducted a Public Health System database retrospective review of TC incident cases from 2000 to 2020 in the Balearic Islands region and evaluated age-standardized incidence rate (ASIR), age at diagnosis, gender distribution, tumor size and histological subtype, mortality rate (MR), and cause of death. Estimated annual percent changes (EAPCs) were also evaluated and data from the 2000–2009 period were compared to the 2010–2020 period when neck ultrasound (US) was routinely performed by clinicians at Endocrinology Departments.

**Results:**

A total of 1387 incident cases of TC were detected. Overall, ASIR (×10^5^) was 5.01 with a 7.82% increment in EAPC. A significant increase in the 2010–2020 period was seen for ASIR (6.99 vs 2.82, *P* < 0.001) and age at diagnosis (52.11 vs 47.32, *P* < 0.001) compared to the 2000–2009 period. A reduction in tumor size (2.00 vs 2.78 cm, *P* < 0.001) and a 6.31% increase in micropapillary TC (*P* < 0.05) were also seen. Disease-specific MR remained stable at 0.21 (×10^5^). The mean age at diagnosis for all mortality groups was older than survivors (*P* < 0.001).

**Conclusion:**

The incidence of TC has grown in the 2000–2020 period in the Balearic Islands, but MR has not changed. Beyond other factors, a significant contribution of overdiagnosis to this increased incidence is likely due to changes in the routine management of thyroid nodular disease and increased availability of neck US.

## Introduction

Thyroid cancer (TC) represents 3% of the global incidence of all cancers, with 586,000 new patients estimated in 2020 worldwide (https://gco.iarc.fr/today/home). It is the most frequent endocrine cancer and its global incidence is growing faster than any other type of malignancy (1), regardless of sociodemographic, ethnic, or environmental differences (2). Conversely, mortality rates (MRs) for TC have decreased or remained stable at 0.5 cases per 100,000 person-years, and 5-year survival rates are maintained over 98% of incident cases (3).

Large epidemiological studies have been published in the last decade, addressing the growing incidence of TC in different regions of the world and the potential factors influencing this increasing trend. A wide heterogeneity is observed in the crude incidence and clinical behavior of major histological subtypes of TC, although most studies point to an increase in the incidence of small papillary carcinomas, potentially as a consequence of the increased performance of routine diagnostic image techniques, resulting in overdiagnosis of incidental subclinical cancers that would be unlikely to cause harm in a person’s lifetime (4, 5, 6, 7, 8, 9). Conversely, an increased incidence of bigger and more aggressive tumors has also been described in developed countries (4, 10). In this heterogeneous scenario, accurate epidemiological studies performed at a population level are needed in order to adequate health resources and estimate the specific impact of overdiagnosis of incident TC in routine clinical practice.

In this article, we present a comprehensive epidemiological analysis of the cumulative incidence and trend changes of TC and its histological variants during two decades (2000–2020) in a developed region of Spain, aiming to put this data in context with other Spanish incidence studies performed earlier and with data from epidemiological studies around the world. Additionally, we present data on total and disease-specific MRs in this large cohort of patients with TC. Finally, we have performed an additional analysis to explore the role of changes in the routine management of thyroid nodular disease, that is, performance of neck ultrasound (US) and fine-needle aspiration cytology (FNAC) by clinicians at Endocrinology Departments, as a potential cause of overdiagnosis.

## Material and methods

We conducted a combined retrospective review of electronic health records from Pathology, Surgery, Endocrinology, and Nuclear Medicine Departments from all six public hospitals of the Balearic Islands Public Health System registries. Cases of TC were identified and after anonymization, data on patient demographics, tumor pathology reports, and survival outcomes were obtained. This study covered registered cases among the resident population from January 2000 to December 2020. The database was checked to avoid duplicated entries. The study protocol was reviewed and approved by the Institutional Ethics Committee of the Balearic Islands and was declared of scientific interest.

Study objectives included primarily descriptive data on the age-standardized incidence rate (ASIR) of TC, using data from the world global population age (1211) as a reference, patient gender and age at diagnosis, tumor size and histological subtype, and survival status by last available observation, including the cause of death, which was classified as total mortality, disease-specific, second neoplasia, and other causes. Secondly, longitudinal changes in trends were evaluated using the estimated annual percentage changes (EAPCs) of ASIR, mean age at diagnosis, size and histological subtype, and cumulative disease-specific MR, aiming to evaluate potential differences throughout the study period. As for other parameters included in this study, ASIR is in an upward trend when the mean EAPC and the lower boundary of the 95% CI are positive and in a downward trend when the mean EAPC and the upper boundary of the 95% CI are negative.

Additionally, since 2010, routine neck US and FNAC of suspicious thyroid nodules have been almost fully incorporated by most Endocrinology Departments in the Balearic Islands public hospitals. Aiming to explore the influence of this relevant change in the management of thyroid nodular disease, that is, as a potential source of overdiagnosis, we searched for differences in all the variables included in this study comparing data from the 2000–2009 period to those of 2010–2020.

## Results

### Thyroid cancer incidence and patients’ demographics

**Figure 1:**
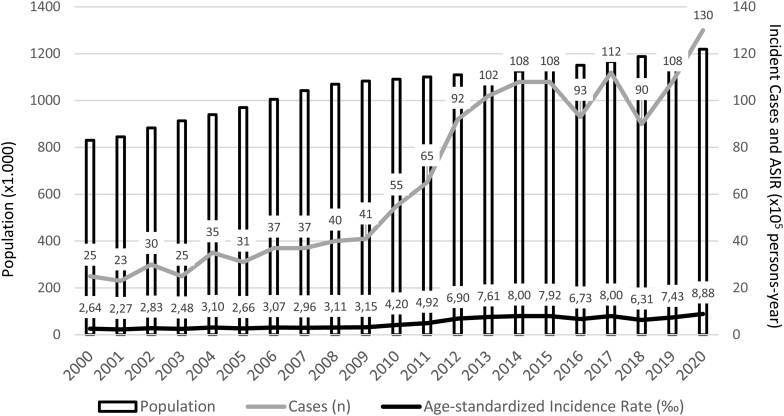
Incident cases and cumulative age-standardized incidence rate (ASIR) of thyroid cancer in the Balearic Islands from 2000 to 2020.

Figure 1 shows incident cases and cumulative ASIR of TC per 100,000 person-years in the Balearic Islands Public Health System registries from 2000 to 2020. Table 1 shows ASIR, mean age at diagnosis, and EAPCs for the whole study period and for pre-specified time frames of 2000–2009 and 2010–2020, respectively. A total of 1387 cases were identified throughout 21 years (77.43% females) with a mean ASIR of 5.01 cases per 100,000 person-years (s.d., 2.36). ASIR of TC changed significantly from 2.82 cases/100,000 person-years (s.d., 0.30) during the 2000–2009 period, to 6.99 (s.d., 2.36) in the 2010–2020 period (147% increase; *P* < 0.001). Gender-specific increases in ASIR were also significant (146% for females and 150% for males; *P* < 0.001 for both comparisons). Mean EAPC of ASIR showed an uptrend of 7.82% (95% CI, 0.36–15.28) for the total cohort and 8.99% (95% CI, 1.53–16.45) for female patients, during the whole study period. During the second period of 2010–2020, only total cohort EAPC of ASIR showed an uptrend of 17.21% (95% CI, 6.22–28.20), with no significant trends for gender-specific or first period (2000–2009) EAPCs.

**Table 1:** Total and gender-specific age-standardized incidence rate, mean age at diagnosis, and trends for thyroid cancer during the 2000–2020 period and during the 2000–2009 vs the 2010–2020 periods.

	Incident cases, *n* (%)	ASIR (mean ± s.d.)	EAPC^b^ (mean; 95% CI)
**2000–2020 period**			
Total incident cases, *n* (%)	1387	5.01 (±2.36)	7.82% (0.36 to 15.28)^c^
Females	1074 (77.43%)	7.76 (±4.39)	8.99% (1.53 to 16.45)^c^
Males	313 (22.57%)	2.25 (±1.25)	0.93% (−6.53 to 8.39)
Total age at diagnosis, years (mean ± s.d.)	49.83 (±3.55)		0.30% (−3.29 to 3.89)
Females	49.40 (±2.94)		0.40% (−3.13 to 3.93)
Males	52.82 (±6.83)^a^		−0.17% (−2.84 to 2.50)
**2000–2009 Period**			
Total incident cases, *n*	324	2.82 (±0.30)	1.22% (−5.51 to 7.95)
Females, *n* (%)	254 (78.4%)	4.39 (±0.77)	7.37% (−2.38 to 17.12)
Males, *n* (%)	71 (21.6%)	1.26 (±0.43)	15.52% (−204.3 to 51.47)
Total age (mean ± s.d.), years	47.32 (±2.61)		−0.12% (−6.32 to 6.08)
Female (mean ± s.d.)	47.39 (±1.75)		−1.38% (−5.1 to 2.34)
Male (mean ± s.d.)	49.64 (±7.8)		2.45% (-13.91 to 18.81)
**2010–2020 period**			
Total incident cases, *n*	1,063	6.99 (±2.36)^b^	17.21% (6,22 to 28.20)^c^
Females, *n* (%)	821 (77.23%)	10.82 (±3.64)^b^	9.73% (−1.26 to 20.72)
Males, *n* (%)	242 (22.77%)	3.16 (±1.31)^b^	−0.71% (−18.02 to 24.16)
Total age (mean ± s.d.), years	52.11 (±2.6)^b^		1.35% (−3.51 to 4.77)
Female (mean ± s.d.)	51.23 (±2.6)^b^		1.29% (−2.74 to 4.36)
Male (mean ± s.d.)	55.72 (±4.38)^ a^		−0.89% (−6.74 to 6.26)

^a^*P* < 0.05; ^b^*P* < 0.001 for the comparison of age at diagnosis (Student’s t-test) and for the comparisons of ASIR (Wilcoxon test) between period 2000–2009 vs 2010–2020 ; ^c^Upward trend, mean EAPC and lower boundary of 95% CI are positive.

ASIR, age-standardized incidence rate per 100,000 person-years; EAPC, estimated annual percent changes.

Age at diagnosis was normally distributed (Supplementary Fig. 1A and B, see section on supplementary materials given at the end of this article), with a mean of 49.83 (s.d., 3.55) years old for the total cohort, and with significantly older male patients compared to females (52.82 s.d., 6.83 vs 49.40 s.d., 2.94; *P* < 0.05). Mean age at diagnosis in the total cohort significantly increased from 47.32 (DE 2.61) years old during the 2000–2009 period to 52.11 (DE 2.60) years old during the 2010–2020 period (*P* < 0.001). Gender-specific increases in mean age at diagnosis were also significant for both female patients (47.39, s.d., 1.75 vs 51.23, s.d., 2.6, *P* < 0.001) and males (49.64, s.d., 7.8 vs 55.72, s.d., 4.38, *P* < 0.05) when 2000–2009 period was compared to 2010–2020. In neither case did EAPCs reveal significant trends in mean age at diagnosis for total or gender-specific groups.

Supplementary Tables 1, 2 and 3 show incident cases of TC, crude incidence and mean age at diagnosis for total and gender-specific populations, respectively, from 2000 to 2020 in the Balearic Islands Public Health Registries.

### Tumor Histological Subtypes and Size

**Figure 2:**
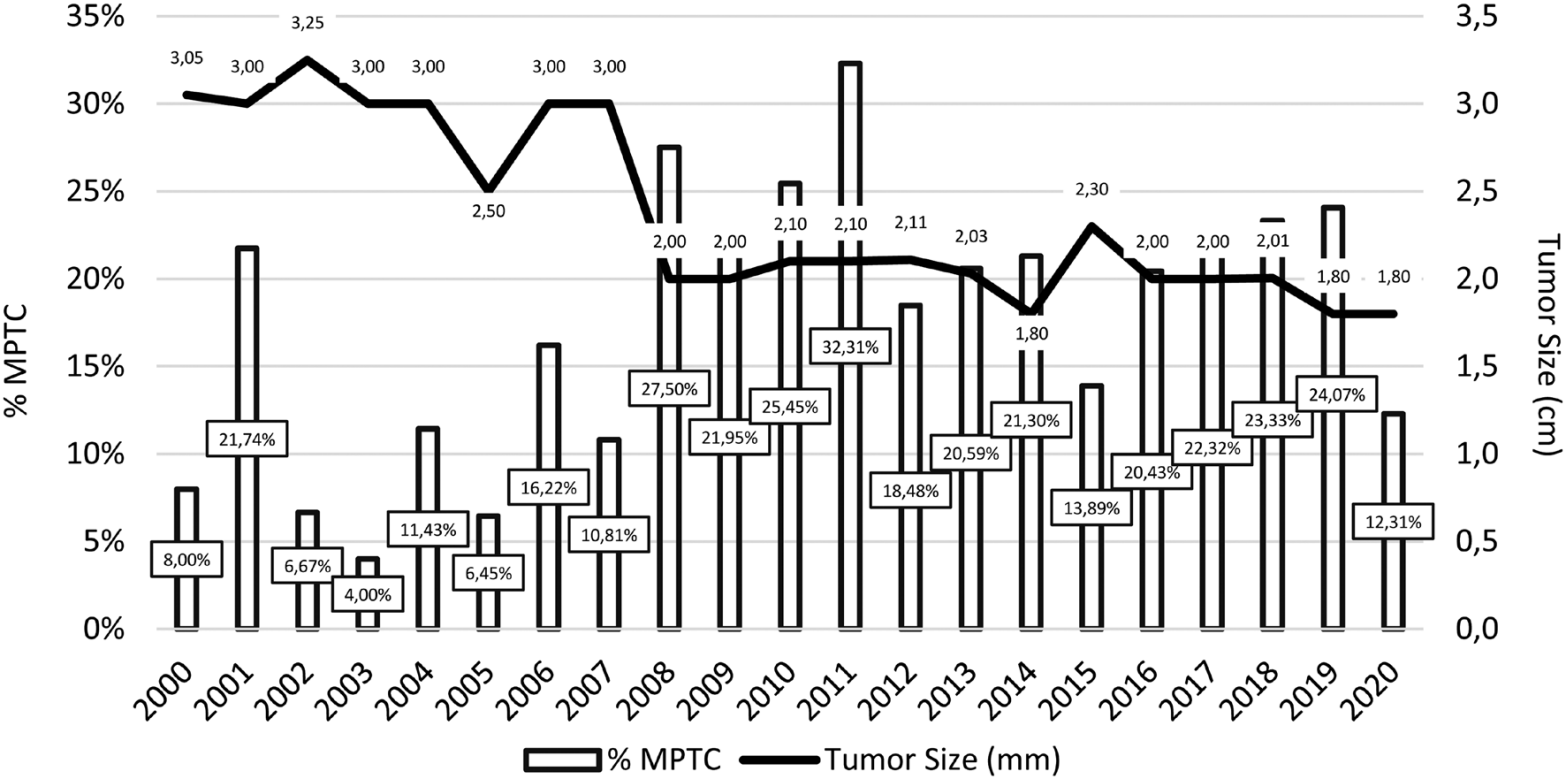
Cumulative percentage of micropapillary thyroid cancer (MPTC) and mean tumor size of thyroid cancer in the Balearic Islands from 2000 to 2020.

Figure 2 shows the cumulative incidence of micropapillary TC (MPTC) and the mean size of TC from 2000 to 2020. Table 2 shows the distribution of TC histological subtypes and size, according to pathology reports, and trends for the whole study period and for the pre-specified time frames of 2000–2009 and 2010–2020, respectively. Papillary TC (excluded MPTC) was the most frequent subtype accounting for 66.14% of cases, followed by MPTC (18.98%), follicular TC (10.85%), medullary TC (2.53%), and poorly differentiated and anaplastic TC (combined 1.5%), respectively. No trends in percent distribution were found for any of the TC histological subtypes, either during the whole study period or within the pre-specified periods of 2000–2009 and 2010–2020. There was a significant 6.31% increase in the percentage of MPTC (20.51% vs 14.20%; *P* < 0.05) in the 2010–2020 period, compared to 2000–2009. The mean tumor size during the whole study period was 2.37 cm (s.d., 0.51). Tumor size showed no trend, neither for the 21 years period nor for the 2000–2009 or 2010–2020 periods, but a significant decrease was observed when mean tumor size was compared between 2000–2009 and 2010–2020, respectively (2.78 cm, s.d., 0.45 vs 2.00 cm, s.d., 0.16; *P* < 0.001).

**Table 2:** Distribution of histological subtypes and size of thyroid cancer (TC) and trends during the 2000–2020 period and during the 2000–2009 vs the 2010–2020 period.

	Incident cases, *n* (%)	EAPC (95% CI)
**2000–2020**		
Histological subtype		
PTC, total	917 (66.14%)	−2.65% (−6.51 to 1.21)
Aggressive histology^a^	77 (5.55%)	0.49% (−1.3 to 2.28)
Micro-PTC	264 (18.98%)	0.88% (−2.83 to 4.59)
Follicular TC	151 (10.85%)	0.15% (−2.58 to 2.88)
Medullary TC	35 (2.53%)	−0.03% (−1.28 to 1.22)
Poorly differentiated TC	10 (0.75%)	0.0% (−0.71 to 0.71)
Anaplastic TC	10 (0.75%)	0.0% (−0.8 to 0.8)
TC mean size (±s.d.), cm	2.37 (±0.51)	−3.04% (−8.31 to 2.23)
**2000–2009**		
Histological subtype, *n* (%)		
PTC, total	213 (65.74%)	−2.57% (−6.96 to 1.82)
PTC, aggressive histology^a^	15 (4.63%)	−0.06% (−2.48 to 2.36)
Micro-PTC	46 (14.20%)	−2.67% (−9.3 to 3.96)
Follicular TC	43 (13.27%)	0.61% (−4.07 to 5.29)
Medullary TC	11 (3.4%)	−0.06% (−2.65 to 2.53)
Poorly differentiated TC	3 (0.93%)	0.0% (−1.39 to 1.39)
Anaplastic TC	3 (0.93%)	0.0% (−1.66 to 1.66)
TC mean size (±s.d.), cm	2.78 (±0.45)	−6.54% (−16.08 to 3.72)
**2010–2020**		
Histological subtype, *n* (%)		
PTC, total	704 (66.23%)	−2.73% (−9.05 to 3.59)
PTC, aggressive histology^a^	62 (5.83%)	0.93% (−1.74 to 3.6)
Micro-PTC	218 (20.51%)^b^	1.01% (−3.12 to 5.14)
Follicular TC	108 (10.15%)	−0.04% (−3.29 to 3.21)
Medullary TC	24 (2.26%)	−0.0% (−0.89 to 0.89)
Poorly differentiated TC	7 (0.66%)	0.0% (−0.66 to 0.66)
Anaplastic TC	7 (0.66%)	0.0% (−0.56 to 0.56)
TC mean size (±s.d.), cm	2.00 (±0.16)^c^	−2.75% (−7.49 to 1.99)

^a^Aggressive histology included tall cell, columnar cell, diffuse sclerosing, solid/trabecular, and insular variants; ^b^ *P* < 0.05 for micro-PTC incidence (chi-square test) between 2000–2009 and 2010–2020 periods; ^c^ *P* < 0.001 for TC mean size (Student’s *t*-test) between 2000–2009 and 2010–2020 periods.

EAPC, estimated annual percent changes; PTC, papillary thyroid carcinoma; TC, thyroid carcinoma.

### Survival outcomes

**Figure 3:**
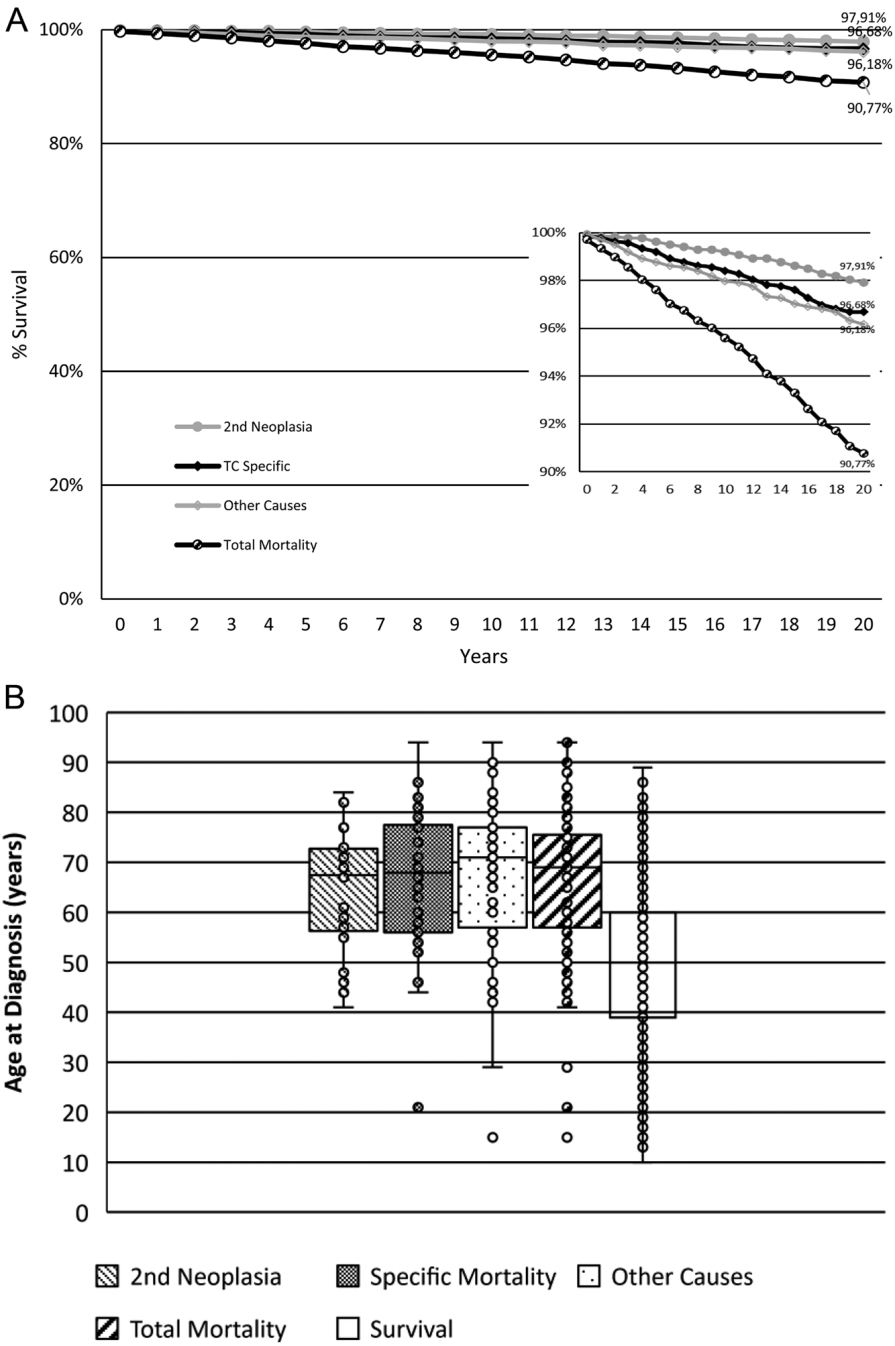
(A) Survival curve by cause of death and last available observation and (B) Box plot indicating age at diagnosis by cause of death in patients with thyroid cancer (TC) in the Balearic Islands from 2000 to 2020.

Figure 3A shows Kaplan–Meier survival curves by total mortality and different causes of death, including second neoplasia, TC disease-specific, and other causes of death, in patients with TC during the 2000–2020 period. Figure 3B shows mean with interquartile range and s.d. for age at diagnosis by total and specific causes of death and for survivors. Tables 3 and 4 show fatality index (FI) (dead cases among incident cases) and MR (dead cases per 100,000 person-years) at 5, 10, and 20 years. FI after 21 years of follow-up was 9.22% (4.4% at 10 years, 2.38% at 5 years) and the overall survival rate was 90.77%, while mean MR during follow-up was 0.58 (0.60 at 10 years, 0.57 at 5 years). The main cause of death, affecting 3.82% of patients at 20 years, was not oncological (other causes), followed by TC disease-specific (3.31%) and second neoplasia (2.09%). The mean age at diagnosis was not different among specific causes of death, and all mortality groups had an older mean age at diagnosis when compared to survivors (*P* < 0.001 for all comparisons). Trends in disease-specific MR were evaluated by EAPC in patients with a minimum follow-up of 5 years, that is, from 2000 to 2015. We did not find a significant trend in the EAPC of MR, neither during the 21 years follow-up nor during the 2000–2009 or 2010–2015 periods. Additionally, we found no differences in mean MR when the 2000–2009 period was compared to the 2010–2015 period (0.21 ± 0.09 vs 0.19 ± 0.07; ns).

**Table 3:** Total and cause-specific mean age at diagnosis, fatality index, and mortality rates in patients with thyroid cancer from 2000 to 2020.

	*n* (%)	Mean age at diagnosis (±s.d.)	Fatality index^a^ (%)			Mortality rate^b^ (×10^5^)		
Cause of death			5 year	10 year	20 year	5 year	10 year	20 year
Second neoplasia	29 (2.09%)	64.04 ± 11.66^c^	0.36%	0.79%	2.09%	0.10	0.12	0.13
Other causes	53 (3.82%)	67.05 ± 15.15^c^	1.23%	2.02%	3.82%	0.25	0.27	0.24
TC-specific	46 (3.31%)	65.34 ± 15.93^c^	0.79%	1.49%	3.31%	0.20	0.21	0.21
Total mortality	128 (9.22%)	65.84 ± 14.77^c^	2.38%	4.4%	9.22%	0.57	0.60	0.58
Survivors	1259 (90.77%)	49.23 ± 14.53						

^a^Fatality index expressed as percent value (%) of incident cases; ^b^Mortality rate expressed as number of deaths by 100,000 person-years; ^c^ *P* < 0.001 compared to survivors (ANOVA test).

**Table 4:** Mean cumulative disease-specific fatality index and mortality rate with estimated annual percent changes (EAPCs) in patients with thyroid cancer from 2000 to 2015 and comparison between 2000–2009 and 2010–2015.

	Mean fatality index^a^ (%) (±s.d.)	EAPC (95% CI)	Mean cumulative mortality rate^b^ (x10^5^) (±s.d.)	EAPC (95% CI)
2000–2015	0.062 ± 0.01	−6.65% (−10.83 to −2.47)^d^	0.22 ± 0.08	14.4% (−20.15 to 48.43)
2000–2009	0.067 ± 0.006	−2.31% (−9.66 to 5.04)	0.21 ± 0.09	14.76% (−40.42 to 69.94)
2010–2015	0.048 ± 0.007^c^	−7.49% (−9.14 to −5.84)^d^	0.19 ± 0.07	13.63% (−19.14 to 46.4)

^a^Fatality index expressed as percent value (%) of total cohort; ^b^Mortality rate expressed as number of deaths by 100,000 person-years; ^c^ *P* < 0.001 compared to 2000–2009 (Wilcoxon test); ^d^Downward trend.

Supplementary Table 4 shows incident mortality by mortality groups and cumulative disease-specific mortality rates during the study period.

## Discussion

In this article, we have presented cumulative and age-standardized incidence of TC in the Balearic Islands population, covering a long period of 21 years. Balearic Islands represents a high-income region in Spain, with a well-developed public health system, including six public hospitals covering a total population of 1,219,423 habitants, which has increased by 31.89% (388,995 habitants) during the time span included in this study. ASIR of TC during the whole study period was 5.01 cases per 100,000 person-years, increasing from 2.82 during the first period (2000–2009) to 6.99 during the second period (2010–2020). According to the International Agency for Research on Cancer, worldwide ASIR for TC during 2020 was 6.6 (12), with an estimate of 7.4 for Spain. Other epidemiological studies coincide with these estimations for Western European and high Socioeconomic Index countries (13, 14). A recent publication by Diaz-Soto and colleagues (15) found a five-fold increase in the crude incidence of TC cases in a Northern region of Spain from 4.55 × 10^5^ in 2002 to 21.6 × 10^5^ in 2017 (crude incidence of 10.66 × 10^5^ in our study). Nevertheless, this incidence could be overestimated, as data were obtained from a highly specialized clinic in a tertiary hospital, and thus, a selection bias cannot be excluded. Finally, a former publication by Galofré (16), evaluated previous studies on TC incidence from different regions in Spain, with a pooled increase in gender-specific crude incidence up to 30 and 10 cases per 100,000 person-years by 2010 in females and males, respectively.

In our study, the annual incidence of TC increased at an average rate of 7.82% per year during the whole 21 years period, indicating a linear increase in the incidence of this disease, which largely coincides with other estimations from Western countries (13, 14, 15, 16, 17, 18). Nevertheless, most of this increase did take place during the second period of time (2010–2020), coinciding with the incorporation of routine performance of neck US and FNAC by most Endocrine Departments of the Balearic Islands Public Health System (five of six centers). In this setting, a potential overdiagnosis of indolent cases cannot be excluded, and this is further supported by some findings: first, a significant reduction in the size of tumors (indicating an increased number of indolent, incidentally discovered nodules) and older patients’ age at diagnosis (as older age is associated to higher prevalence of thyroid nodular disease) during this second period; second, a significant increase (6.31%) in the incidence of MPTC. Indeed, taken together all these changes might reflect an increased number of thyroid nodes undergoing cytology as a consequence of the ‘learning curve’ of physicians involved in patient care. In this sense, an increased number of thyroid cytology studies was registered in our institution from 2010 to 2015 (data on file), with a subsequent reduction from 2016 onwards, indicating perhaps a more accurate selection of cases undergoing FNAC. Additionally, the availability of neck US at the physician’s office is associated with an increased number of explorations and potentially, to a growing number of thyroid surgeries in patients with multinodular goiter, which in turn, would increase the incidence of occult PTC. This important factor could explain the significant reduction in tumor size observed, despite the specific recommendation against the performance of routine cytology in small thyroid nodules (19), and the observed increase in patients’ age at diagnosis in the 2010–2020 period, as the prevalence of multinodular disease of the thyroid increases with age. Furthermore, the 2014 World Cancer Report stated that, among the new cases of thyroid carcinoma, >50% are MPTC (20), being the rapid development of imaging diagnosis technology is one of the main reasons behind the overdiagnosis of TC. In agreement with this, all epidemiological studies in Spain, including ours, have pointed to a significant increase in the incidence of MPTC (16), and overall prevalence of MPTC in published series in Spain is estimated around 15–20% (21).

In line with other studies evaluating histological subtypes of TC (14), we did not find a significant change in the distribution of papillary (including aggressive variants), follicular, medullary, poorly differentiated, or anaplastic subtypes of TC. As mentioned earlier, only MPTC experienced a statistically significant 6.31% increase in incidence (from 14.20 to 20.51%) in the 2010–2020 period. Notably, the absence of a trend toward an increased incidence of MPTC in this second period of the study might reflect a more conservative approach in the management of thyroid nodular disease in recent years (19). Other publications have addressed a reduction in the global incidence of papillary TC possibly as a consequence of rising awareness of problems caused by overtreatment (22) and the prospect that patients are more clearly advised of the benefits and harms in treating low-risk microtumors.

We evaluated long-term survival, causes of death, and MRs and trends in this cohort of patients with TC. Overall survival at 5 years (97.62%) and disease-specific mean MR (0.20 per 100,000 person-years) were absolutely in line with reported rates around the world, which are far less influenced by geographical heterogenicity in incidence (3, 4, 13, 21, 23). Two previous publications have evaluated disease-specific mortality in TC in Spain; from 1975 to 2001, the crude MR in Spain was reduced to 0.5 (×10^5^) according to the National Centre for Epidemiological Studies (24), and more recently, another study in the Southern province of Granada showed a stable crude MR of 0.4 for men and 0.5 for women (×10^5^) without trend changes, during the 1985–2013 period (25). In our study, MR remained stable at 0.20 (×10^5^), but at the same time, in line with other studies, we found a reduction in the disease-specific FI which was more pronounced in the 2010–2015 period. Again, the combination of a stable population-based MR with a reduction in incidence-based mortality index clearly supports a significant role in the overdiagnosis of subclinical indolent disease in the observed increase in TC incidence, although an early diagnosis of more aggressive cases and as a consequence, an improvement in their prognosis, cannot be excluded. TC disease-specific was not the main cause of death among patients with TC in our study, reflecting the low aggressiveness of most TCs, although some authors have pointed a true increase in incidence-based mortality (26) due to potential environmental factors affecting a subset of fast-growing TCs, which we did not observe in our study. Of note, the age at diagnosis in all mortality groups was older than that of the survivors, confirming the well-established role of age as an independent predictor of mortality risk in TC (19).

Our study has some limitations and strengths. Among the first, the Balearic Islands is a wealthy region with a robustly developed private insurance-based health system, so underestimation of true incidence cannot be fully excluded as we might have missed incident cases attended in the private setting. Nevertheless, we observed a not negligible number of cases initially diagnosed in the private setting but then transferred to the public health system, probably due to the oncological nature of the disease. Additionally, we performed an additional exploratory evaluation of TC cases followed in the private sector and found that virtually all of them were registered in our database. Conversely, the main strength of this study is the multidisciplinary design of the database, involving all departments involved in health care for patients with TC, in all the public Health System hospitals of the Balearic Islands, virtually covering the whole region’s population and thus reducing the risk for selection bias or missing data, further supported by the large coincidence of TC incidence found in our study with previously reported data.

In conclusion, the ASIR of TC in our region during the 2000–2020 period has experienced an increase that parallels that seen in other parts of the world with a similar sociodemographic background. Nevertheless, overdiagnosis derived from the adoption of neck US and FNAC of suspicious thyroid nodes by clinicians attending Endocrinology Units is likely to have played a significant role in the abrupt increase in TC incidence observed in the second period of this study. We did not observe a significant change in the distribution of histological subtypes of TC, with the exception of a moderate increase in MPTC, along with older age at diagnosis in the 2010–2020 period that could further support the role of overdiagnosis. Additionally, we have reported data on total and disease-specific mortality which remained stable despite the increased TC incidence, consistent with reported worldwide trends.

Finally, our study should serve as a call to attention, based upon the widespread use of image techniques by clinicians at the Office, in order to adhere to evidence-based medicine endorsed by current guidelines for the management of thyroid nodular disease, avoiding overdiagnosis and its inherent risk of turning healthy people into patients, exposing them to unnecessary harms and lifelong treatments. Conversely, the increased accessibility of diagnostic techniques should enhance our ability to detect and offer early and well-balanced management options for potentially life-threatening thyroid tumors.

## Supplementary Material

Supplementary Material

## Declaration of interest

The authors declare that there is no conflict of interest that could be perceived as prejudicing the impartiality of the research reported.

## Funding

This work did not receive any specific grant from any funding agency in the public, commercial, or not-for-profit sector.
