# Supplementary material for: Age-standardized incidence, mortality rate, and trend changes of thyroid cancer in the Balearic Islands during the 2000–2020 period: a population-based study
Source: Eur Thyroid J. 2023 Apr 21;12(3):e220183. doi: 10.1530/ETJ-22-0183 (PMC10160559; doi:10.1530/ETJ-22-0183)

Supplementary Material

Table S1. Crude Incidence and Mean Age at Diagnosis for Thyroid Cancer during the 2000-2020 Period in the Balearic Islands Public Health Records.

Year	Population*	Incident Cases, n	Percent	Crude Incidence, (x100.000)	Mean Age at Diagnosis, years
2000	830.428	25	1,80%	3,01	45,32
2001	845.130	23	1,66%	2,72	49,43
2002	883.410	30	2,16%	3,40	46,81
2003	912.964	25	1,80%	2,74	46,29
2004	940.124	35	2,52%	3,72	47,54
2005	970.288	31	2,24%	3,19	42,78
2006	1.005.184	37	2,67%	3,68	52,26
2007	1.042.855	37	2,67%	3,55	46,43
2008	1.070.164	40	2,88%	3,74	49,65
2009	1.083.683	41	2,96%	3,78	46,75
2010	1.091.656	55	3,97%	5,04	48,07
2011	1.100.715	65	4,69%	5,91	53,09
2012	1.110.115	92	6,63%	8,29	47,63
2013	1.115.841	102	7,35%	9,14	52,19
2014	1.124.972	108	7,79%	9,60	53,61
2015	1.135.527	108	7,79%	9,51	52,32
2016	1.150.935	93	6,71%	8,08	57,49
2017	1.166.923	112	8,07%	9,60	52,54
2018	1.188.220	90	6,49%	7,57	53,26
2019	1.210.725	108	7,79%	8,92	51,66
2020	1.219.423	130	9,37%	10,66	45,32

*Source: National Institute of Statistics at https://ine.es/jaxiT3/Tabla.htm?t=31304. Accessed 31/08/2022.

Table S2. Crude Incidence and Mean Age at Diagnosis for Thyroid Cancer in Female Population during the 2000-2020 Period.

Year	Population*	Incident Cases, n	Percent vs Male Population	Crude Incidence, (x100.000)	Mean Age at Diagnosis, years
2000	418.868	20	80,00%	4,77	50,25
2001	425.993	15	65,22%	3,52	46,80
2002	444.000	26	86,67%	5,86	48,08
2003	458.415	21	84,00%	4,58	47,48
2004	471.245	31	88,57%	6,58	46,68
2005	485.509	21	67,74%	4,33	45,06
2006	501.617	25	67,57%	4,98	49,54
2007	518.832	30	81,08%	5,78	46,37
2008	531.524	33	82,50%	6,21	48,63
2009	538.545	31	75,61%	5,76	45,03
2010	543.069	41	74,55%	7,55	46,85
2011	548.122	55	84,62%	10,03	51,16
2012	553.557	82	89,13%	14,81	46,69
2013	557.211	73	71,57%	13,10	52,52
2014	562.431	85	78,70%	15,11	53,63
2015	567.982	87	80,56%	15,32	52,24
2016	575.631	72	77,42%	12,51	55,55
2017	583.467	79	70,54%	13,54	51,42
2018	593.797	68	75,56%	11,45	52,09
2019	604.862	85	78,70%	14,05	51,03
2020	609.611	94	72,31%	15,42	50,30

*Source: National Institute of Statistics at https://ine.es/jaxiT3/Tabla.htm?t=31304. Accessed 31/08/2022.

Table S3. Crude Incidence and Mean Age at Diagnosis for Thyroid Cancer in Male Population during the 2000-2020 Period.

Year	Population*	Incident Cases, n	Percent	Crude Incidence, (x100.000)	Mean Age at Diagnosis, years
2000	411.560	5	20,00%	1,21	34,60
2001	419.137	8	34,78%	1,91	56,50
2002	439.410	4	13,33%	0,91	49,75
2003	454.549	4	16,00%	0,88	51,00
2004	468.878	4	11,43%	0,85	54,25
2005	484.779	10	32,26%	2,06	38,22
2006	503.568	12	32,43%	2,38	58,80
2007	524.023	7	18,92%	1,34	46,71
2008	538.640	7	17,50%	1,30	54,42
2009	545.138	10	24,39%	1,83	52,10
2010	548.586	14	25,45%	2,55	51,64
2011	552.593	10	15,38%	1,81	63,70
2012	556.558	10	10,87%	1,80	55,30
2013	558.631	29	28,43%	5,19	51,37
2014	562.541	23	21,30%	4,09	53,52
2015	567.545	21	19,44%	3,70	52,66
2016	575.304	21	22,58%	3,65	64,14
2017	583.456	33	29,46%	5,66	55,27
2018	594.424	22	24,44%	3,70	57,09
2019	605.863	23	21,30%	3,80	53,95
2020	609.812	36	27,69%	5,90	54,27

*Source: National Institute of Statistics at https://ine.es/jaxiT3/Tabla.htm?t=31304. Accessed 31/08/2022.

Table S4. Incident Mortality by Mortality Groups in Patients with Thyroid Cancer in the Balearic Islands during the 2000-2020 period.

Year	Disease-specific	Other Causes	2nd Neoplasia	Total Incident Cases	Cumulative Disease-specific Mortality Rate† (%)
2000	2	1	1	25	0,080
2001	1	3	1	23	0,063
2002	2	3	0	30	0,064
2003	1	4	1	25	0,058
2004	3	4	0	35	0,065
2005	2	2	2	31	0,065
2006	4	2	2	37	0,073
2007	2	1	1	37	0,070
2008	2	2	2	40	0,067
2009	1	3	0	41	0,062
2010	2	3	1	55	0,058
2011	2	1	2	65	0,054
2012	3	2	2	92	0,050
2013	3	6	0	102	0,047
2014	1	1	2	108	0,042
2015	2	3	2	108	0,039
2016	5	2	2	93	0,040
2017	4	1	3	112	0,040
2018	2	2	1	90	0,038
2019	2	5	2	108	0,037
2020	0	2	2	130	0,033

† Cumulative Mortality rate expressed as percent value (%) of total cohort.

Table S5. Disease-specific Population-based and Cumulative Mortality Rate in Patients with Thyroid Cancer in the Balearic Islands during the 2000-2020 period.

Year	Disease-specific Mortality Incident Cases	Balearic Islands Population	Population-based Mortality Rate ($x10^5$)	Total Incident Cases	Cumulative Disease-specific Mortality Rate[†] (%)
2000	2	830,428	0.24	25	0,080
2001	1	845,130	0.12	23	0,063
2002	2	883,410	0.23	30	0,064
2003	1	912,964	0.11	25	0,058
2004	3	940,124	0.32	35	0,065
2005	2	970,288	0.21	31	0,065
2006	4	1,005,184	0.40	37	0,073
2007	2	1,042,855	0.19	37	0,070
2008	2	1,070,164	0.19	40	0,067
2009	1	1,083,683	0.09	41	0,062
2010	2	1,091,656	0.18	55	0,058
2011	2	1,100,715	0.18	65	0,054
2012	3	1,110,115	0.27	92	0,050
2013	3	1,115,841	0.27	102	0,047
2014	1	1,124,972	0.09	108	0,042
2015	2	1,135,527	0.18	108	0,039
2016	5	1,150,935	0.43	93	0,040
2017	4	1,166,923	0.34	112	0,040
2018	2	1,188,220	0.17	90	0,038
2019	2	1,210,725	0.17	108	0,037
2020	0	1,219,423	0	130	0,033

[†] Cumulative Mortality rate expressed as percent value (%) of total cohort.

Figure S1) Gender-specific and S2) Incident Cases Age Distribution of Patients with Thyroid Cancer during the 2000-2020 Period in the Balearic Islands,

S1)

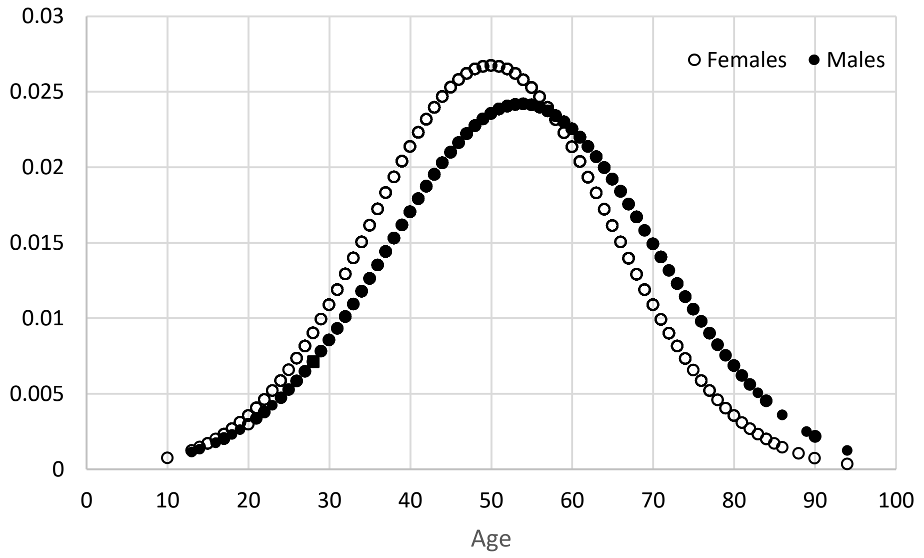

S2)

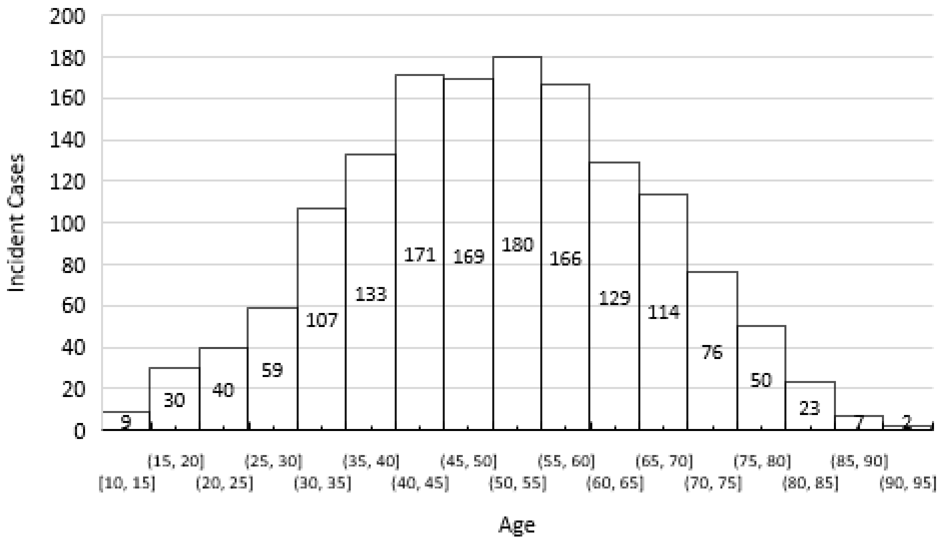

Supplement: Supplementary Material [file supplementary_material.pdf]
